# Removal of Prostate Calcifications Prior to TULSA-PRO With the Aid of Real-Time Ultrasound Imaging: Our Technique and Experience

**DOI:** 10.7759/cureus.92995

**Published:** 2025-09-23

**Authors:** Kyle Johnson, Rahul Mehan

**Affiliations:** 1 Surgery, Midwestern University Arizona College of Osteopathic Medicine, Glendale, USA; 2 Urology, Dignity Health Mercy Gilbert Medical Center, Gilbert, USA

**Keywords:** computed tomography, focal therapy, localized prostate cancer, low-dose ct, prostate calcifications, prostatic calculi, transrectal ultrasound, transurethral ultrasound ablation, tulsa-pro, ultrasound-guided resection

## Abstract

Prostate calcifications can impede ultrasound energy propagation during transurethral ultrasound ablation (TULSA-PRO, Profound Medical Inc., Mississauga, ON, Canada). We instituted a pre-procedure screening protocol using low-dose pelvic computed tomography (CT) with ≤2-mm slices to detect calcifications larger than 3 mm along the planned ablation path; when obstructing deposits are present, we clear them immediately before treatment. Drawing on image-guided principles used in Aquablation (AquaBeam Robotic System, PROCEPT BioRobotics, San Jose, CA, USA), we correlate preoperative sagittal CT with real-time, stepper-mounted transrectal ultrasound (TRUS) to localize targeted prostate tissue for limited bipolar resection, while continuously visualizing the loop position on TRUS. In eight consecutive candidates with obstructive calcifications ≥3 mm, post-procedure low-dose CT confirmed clearance in every case, enabling uninterrupted continuation to the TULSA-PRO workflow. This streamlined, CT-referenced, TRUS-guided technique offers a practical pathway to preserve beam-path integrity without broad tissue debulking. The dataset of eight consecutive candidates with obstructive calcifications ≥3 mm reported here is original and has not been previously published in whole or in part.

## Introduction

TULSA-PRO (Profound Medical Inc., Mississauga, ON, Canada) delivers directional transurethral ultrasound energy under magnetic resonance imaging thermometry and has shown favorable oncologic and functional outcomes in prospective studies, early-phase work, and single-center experience [1-7]. Calcifications situated in the planned energy path can distort acoustic propagation and local heat deposition. Because calcifications are common and increase with age, systematic screening is warranted [8,9]. Our program therefore requires low-dose pelvic CT with ≤2-mm slice thickness to map deposits with a maximum diameter larger than 3 mm; calcifications larger than 3 mm that intersect the planned path are considered an exclusion unless cleared prior to ablation. Device documentation emphasizes avoiding calcifications in the beam path, and simulation work supports this planning principle [10,11]. CT-based methods aid reliable detection of clinically relevant calcifications [12]. The pivotal trial evaluating this technology is also publicly registered [13]. The objective of this report is to describe a reproducible, image-guided clearance technique and our early experience across eight consecutive candidates.

## Technical report

**Figure 1:**
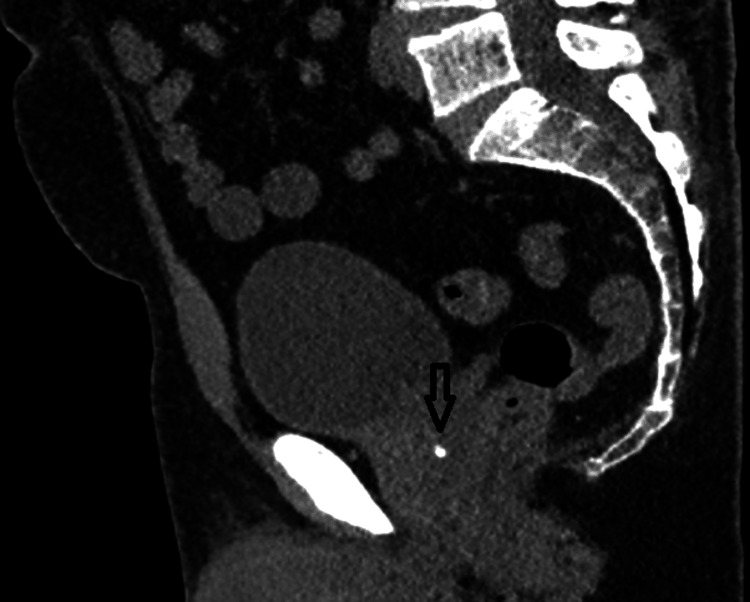
Pre-procedure low-dose CT (sagittal) demonstrating calcification (arrow) within the planned beam path (original image).

All candidates undergo non-contrast, low-dose pelvic CT with ≤2-mm slices, the transverse slice thickness is 0.6mm, and transverse, sagittal, and coronal planes were acquired (Figure 1). Sagittal and axial reconstructions are reviewed to identify any calcification intersecting the planned ultrasound path.

**Figure 2:**
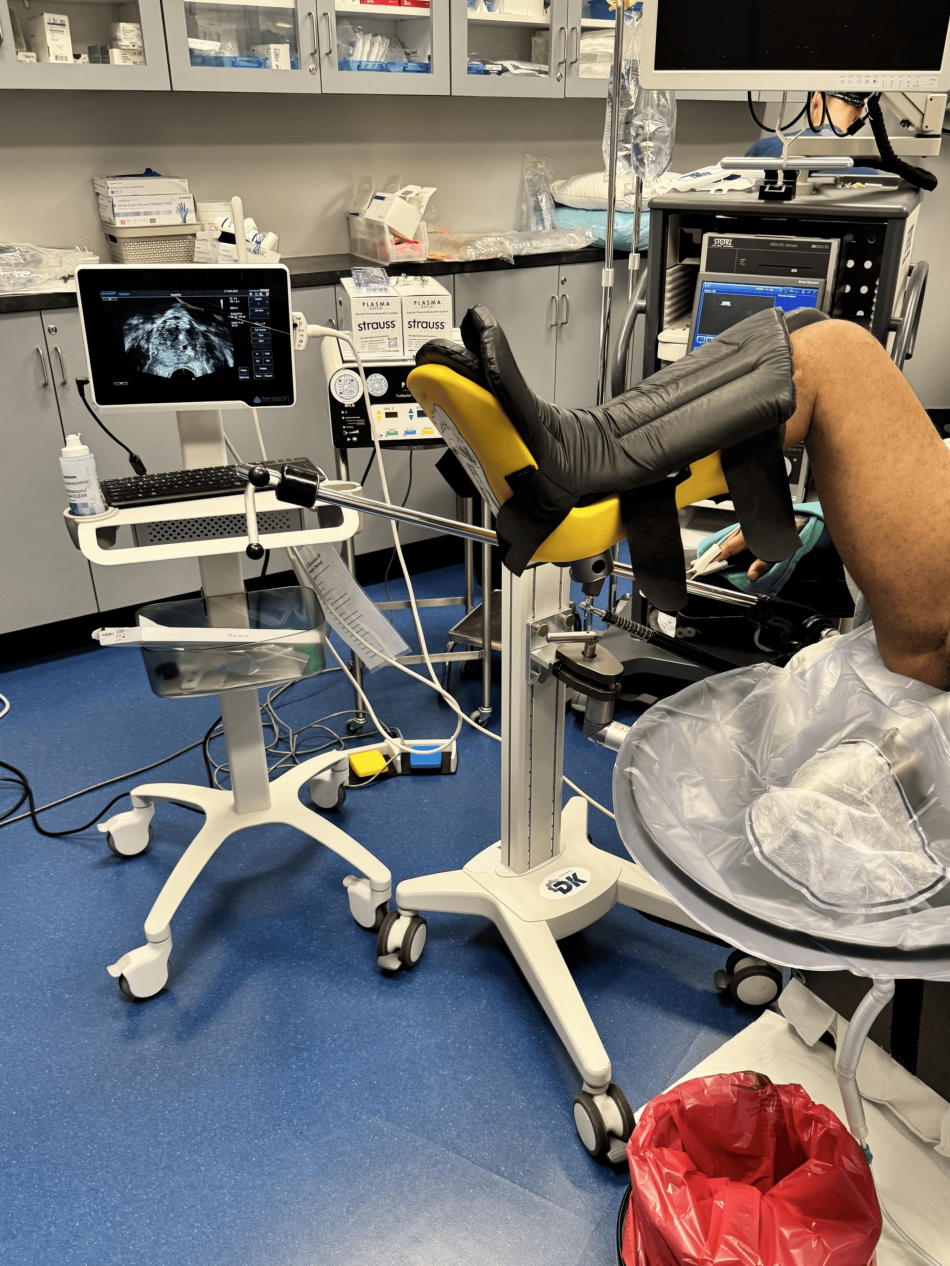
Operating room setup. Stepper-mounted transrectal ultrasound adjacent to the operative field with the resectoscope tower aligned to the surgeon. Source: Authors; no copyrighted material used; permission not required.

The operating room setup is shown in Figure 2. In the lithotomy position, a stepper-mounted transrectal ultrasound (TRUS) probe is positioned adjacent to the field with the resectoscope tower aligned to the surgeon’s view and continuously monitored. Figure 2 depicts probe and stepper orientation.

**Figure 3:**
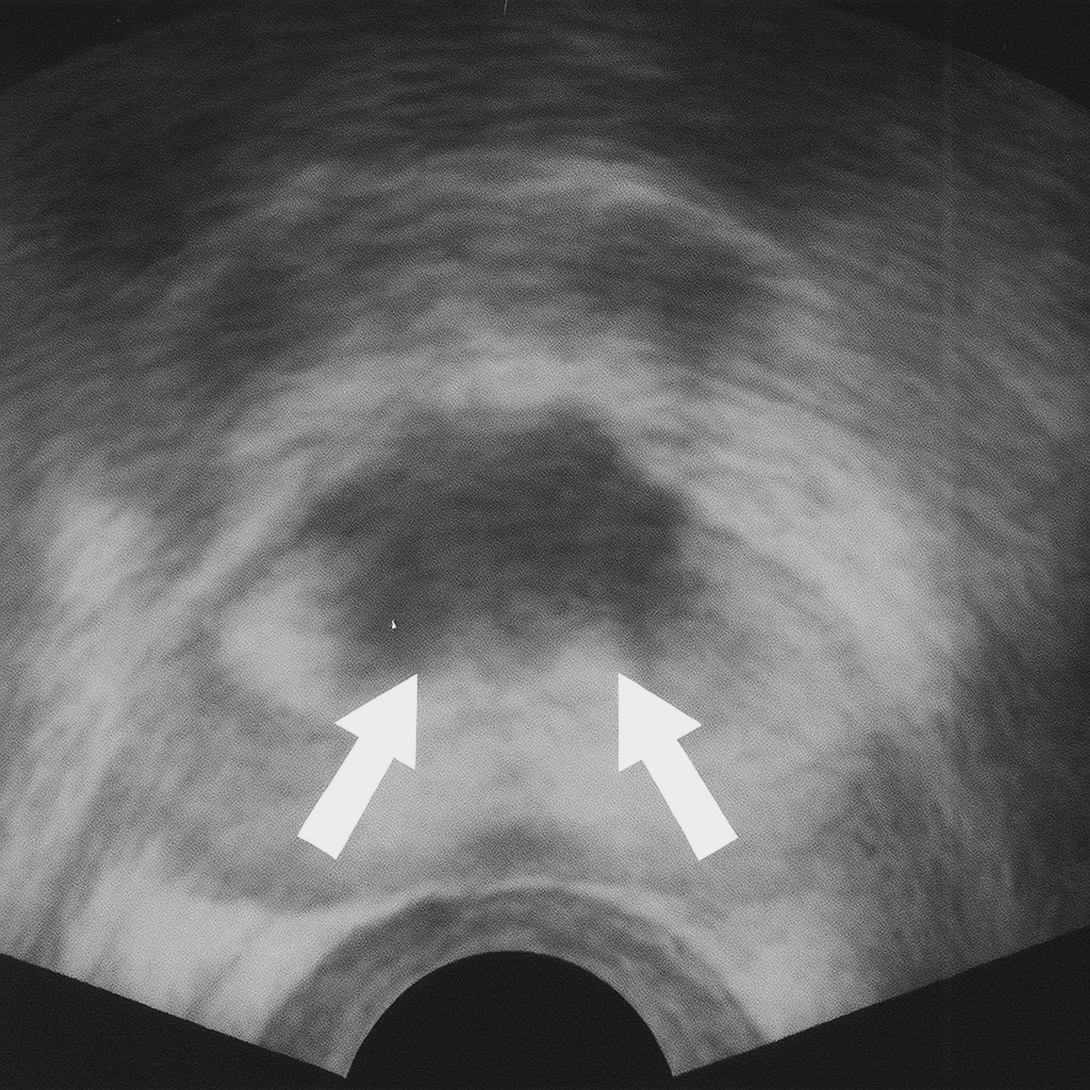
TRUS before resection (sagittal). Hyperechoic focus with posterior acoustic shadowing corresponding to the calcification (digital arrows point to the calcification/shadowing border) mapped on preoperative CT (original image). Source: Authors; no copyrighted material used; permission not required. TRUS: Transrectal ultrasound

TRUS acquisition and targeting are shown in Figure 3. After rectal preparation, 60 mL of surgical lubricant is instilled, with additional lubricant on the TRUS probe tip. The probe is inserted in the sagittal view, kept parallel to the floor, and advanced until 2-3 cm of bladder is visualized. If resistance is encountered, the probe is withdrawn and reinserted with a more posterior trajectory; gentle anterior compression optimizes the window. The view is toggled to transverse to center the prostate from base to mid-gland, then returned to sagittal. Because calcifications are variably echogenic, sagittal TRUS is continuously compared to sagittal CT side-by-side to align the resection target. Figure 3 shows a representative pre-resection TRUS with posterior acoustic shadowing.

**Figure 4:**
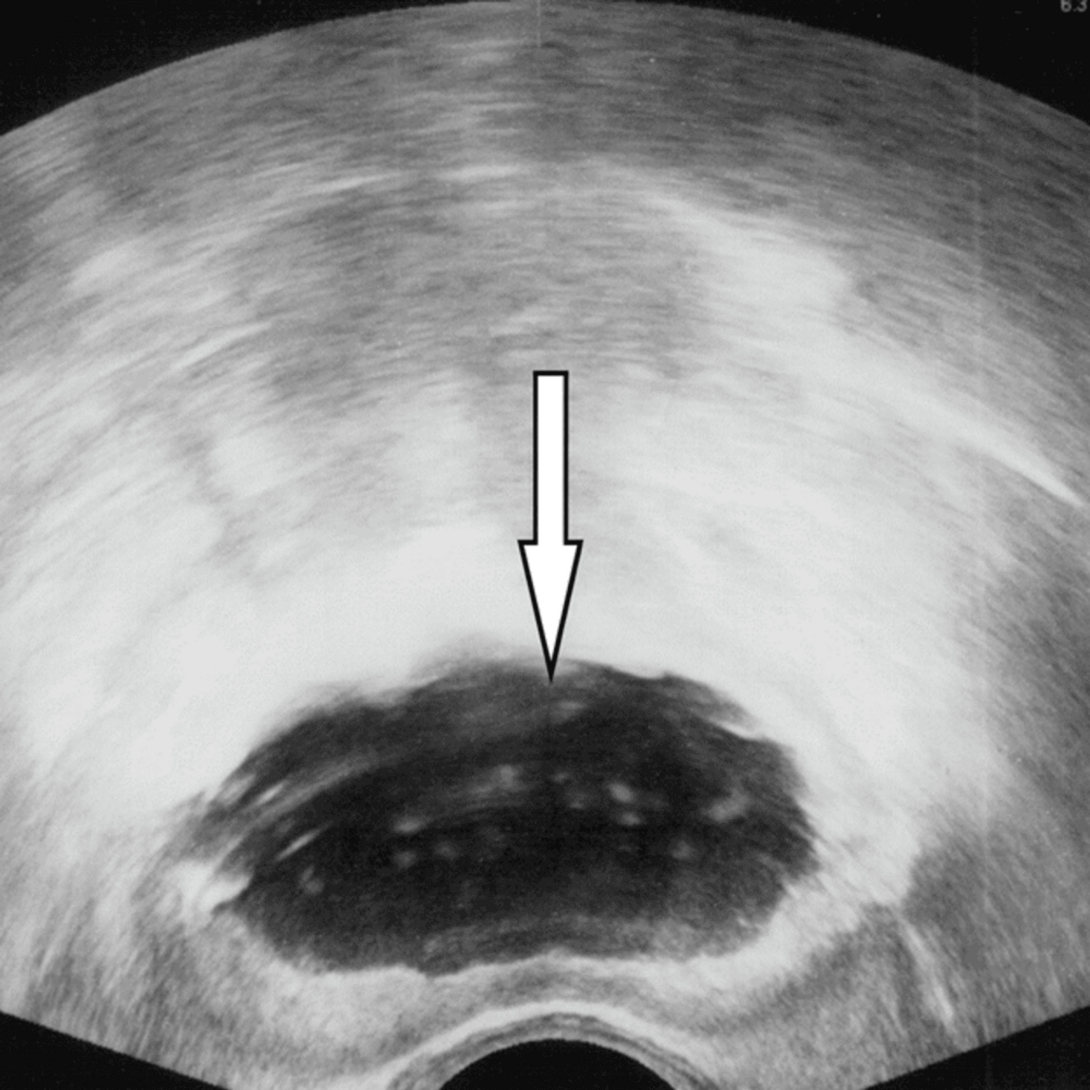
TRUS after targeted clearance complete showing resolution of shadowing and a smooth, symmetric channel with digital arrow marking a smooth symmetric channel (original image). Source: Authors; no copyrighted material used; permission not required. TRUS: Transrectal ultrasound

Targeted resection under live ultrasound is shown in Figure 4. A 24-French bipolar resectoscope is introduced with a visual obturator. Short, controlled cuts remove only the tissue overlying or immediately adjacent to the CT-localized calcification. Live TRUS remains visible throughout to verify loop position relative to the target and capsule and to assess for residual shadowing. Hemostasis is confirmed under normotensive conditions; the bladder and fossa are irrigated to remove chips, and the prostatic channel is reassessed on TRUS in sagittal and transverse planes. A three-way hematuria catheter is then placed under TRUS guidance. Figure 4 presents resolution of shadowing with a smooth, symmetric channel.

**Figure 5:**
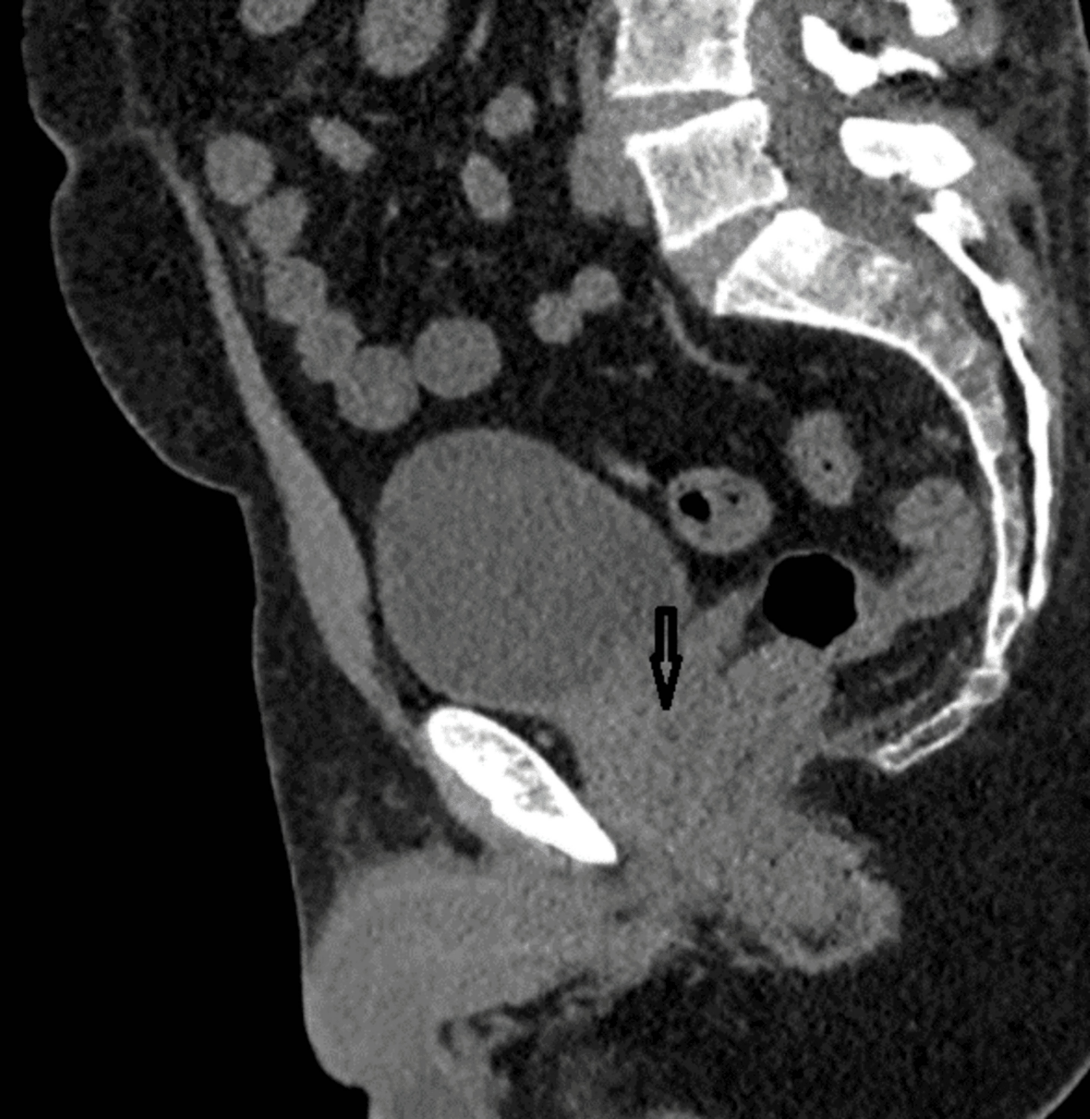
Post-procedure low-dose CT (sagittal) showing clearance of the calcification at the same level (arrow points to the location of previous calcifications), confirming an unobstructed beam path (original image). Source: Authors; no copyrighted material used; permission not required.

Post-procedure verification and series outcome are shown in Figure 5. A repeat low-dose CT is obtained to confirm clearance of the targeted calcification before proceeding with TULSA-PRO and shows the absence of the prior calcification at the same sagittal level. Across eight consecutive candidates in whom preoperative CT revealed calcifications at least 3 mm intersecting the planned beam path, post-resection CT confirmed complete clearance in all cases, permitting uninterrupted continuation to TULSA-PRO. No clearance-step complications were recorded in this series.

## Discussion

Prostatic calcifications are common and clinically relevant when they intersect an ultrasound energy path [8,9]. For transurethral ultrasound ablation, such foci can distort acoustic propagation and heat deposition; this is reflected in manufacturer guidance to avoid calcifications in the beam path and in simulation data demonstrating field perturbation in their presence [10,11]. Our response is pragmatic: screen preoperatively and, when needed, clear the obstruction immediately before ablation.

Low-dose pelvic CT with thin slices can detect small intraprostatic calcifications and support three-dimensional planning of the ablation trajectory [12], although population-specific accuracy measures have not yet been established. Coupling this map with real-time, stepper-mounted TRUS allows limited, targeted resection of only the overlying tissue, immediate confirmation of clearance on ultrasound, and fast post-procedure CT verification. In eight consecutive candidates with obstructing deposits ≥3 mm, this workflow enabled uninterrupted continuation to TULSA-PRO. Maintaining beam-path integrity aligns the procedural setup with studies in which TULSA achieved favorable early cancer control and functional outcomes [1-7]. Across eight consecutive candidates in whom preoperative CT revealed calcifications at least 3 mm intersecting the planned beam path, post-resection CT confirmed complete clearance in all cases, permitting uninterrupted continuation to TULSA-PRO. No clearance-step complications were recorded in this series; however, the small sample size, absence of a control group, and lack of long-term outcomes limit the strength and generalizability of these findings.

## Conclusions

A programmatic workflow consisting of CT screening with ≤2-mm slices to detect deposits larger than 3 mm, exclusion of obstructing calcifications unless cleared, and real-time TRUS-guided limited resection achieved complete clearance in eight consecutive candidates and preserved the downstream TULSA-PRO plan. This practical technique can help maintain beam-path integrity without unnecessary tissue removal. In practice, this approach fits neatly into the existing TULSA-PRO setup and adds minimal time. It also provides a clear, reproducible decision pathway for teams encountering calcifications on pre-procedure imaging. Adoption may be especially helpful for centers early in their TULSA learning curve.
